# The Impact of Metagenomics on Biocatalysis

**DOI:** 10.1002/anie.202402316

**Published:** 2024-04-03

**Authors:** Bethany N. Hogg, Christian Schnepel, James D. Finnigan, Simon J. Charnock, Martin A. Hayes, Nicholas J. Turner

**Affiliations:** ^1^ Department of Chemistry University of Manchester Manchester Institute of Biotechnology 131 Princess Street Manchester M1 7DN UK; ^2^ School of Engineering Sciences in Chemistry, Biotechnology and Health Department of Industrial Biotechnology KTH Royal Institute of Technology AlbaNova University Center 11421 Stockholm SE; ^3^ Prozomix Building 4 West End Ind. Estate Haltwhistle NE49 9HA UK; ^4^ Compound Synthesis and Management Discovery Sciences Biopharmaceuticals R&D AstraZeneca Mölndal 431 50 Gothenburg SE

## Abstract

In the ever‐growing demand for sustainable ways to produce high‐value small molecules, biocatalysis has come to the forefront of greener routes to these chemicals. As such, the need to constantly find and optimise suitable biocatalysts for specific transformations has never been greater. Metagenome mining has been shown to rapidly expand the toolkit of promiscuous enzymes needed for new transformations, without requiring protein engineering steps. If protein engineering is needed, the metagenomic candidate can often provide a better starting point for engineering than a previously discovered enzyme on the open database or from literature, for instance. In this review, we highlight where metagenomics has made substantial impact on the area of biocatalysis in recent years. We review the discovery of enzymes in previously unexplored or ‘hidden’ sequence space, leading to the characterisation of enzymes with enhanced properties that originate from natural selection pressures in native environments.

## Graphical abstract

Metagenomics generally involves isolation and extraction of DNA from various environmental samples, which can then be cloned and expressed into proteins of interest to address difficult chemical reactions in biocatalysis.

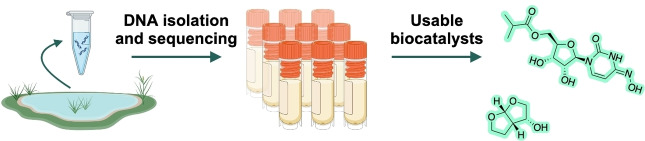

## Metagenomics and its Role in Biotechnology

**Figure 1:**
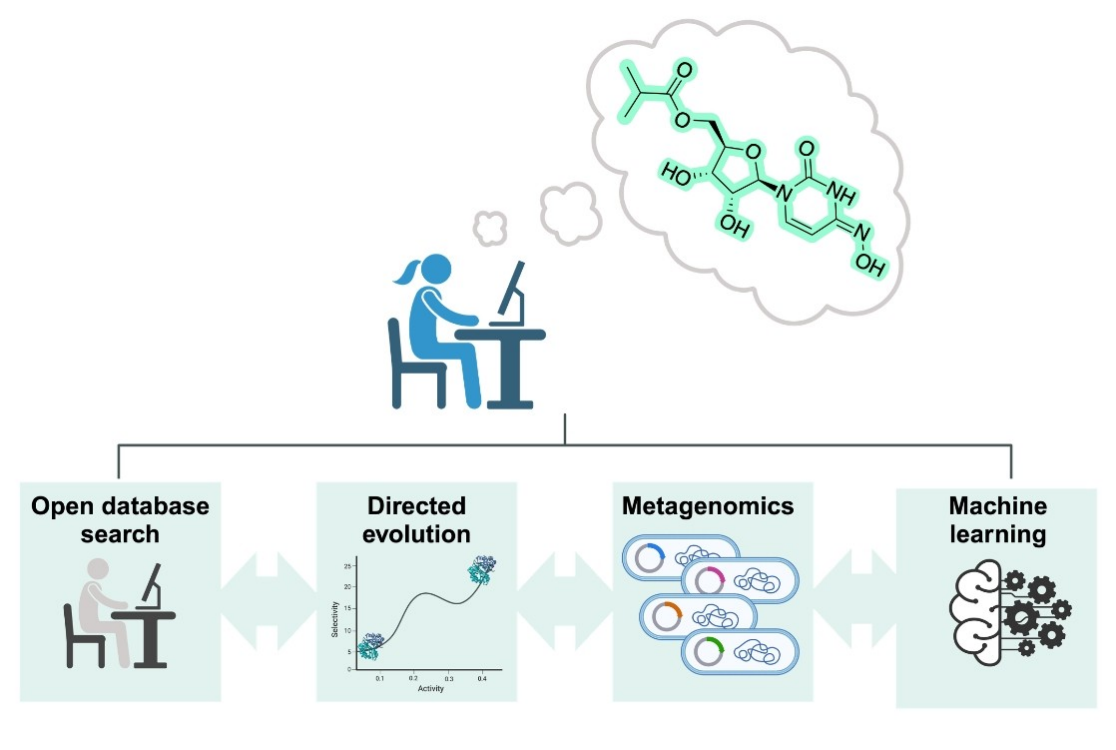
The ‘four pillars’ of metagenomic driven biocatalysis. With the aim of identifying novel biocatalysts for chemical synthesis, researchers explore a combination of (i) open database searching for enzymes of interest; (ii) directed evolution of previously known enzymes; (iii) searching metagenomic databases for novel biocatalysts and (iv) implementing machine learning to predict the most suitable enzyme for the desired transformation.

In recent years, the field of biocatalysis has established itself in the chemical and pharmaceutical sectors as a green alternative to traditional chemistry^[1]^ The advantages of biocatalysis include reactions conducted in aqueous media under mild conditions, and the associated high regio‐ and enantio‐selectivity without the need for cumbersome purification steps.^[2]^ Consequently, the use of biocatalysts in industrial processes is becoming increasingly popular as a result of economic and environmental advantages along with the urgent requirement to create sustainable manufacturing processes for chemical production.(Figure 1).^[3]^ Many companies have begun to commit to the United Nation's Sustainability Goals, striving to meet many of the 17 targets defined by the United Nations.^[4]^ Despite this emerging change in economic culture, the development of viable bioprocesses still encounters several challenges to become a more universally applied technology that outcompetes conventional chemical manufacturing processes in terms of efficiency, productivity, and environmental footprint.

Currently a significant amount of time, effort and cost are associated with the discovery of enzymes for specific applications. In many cases a biocatalyst identified with the desired characteristics is subjected to multiple rounds of protein engineering to evolve the enzyme into a more robust biocatalyst that is tailored for process‐specific conditions. A common caveat of enzyme engineering is that at least trace amounts of starting activity are required for the engineering campaign to succeed. In addition, the lack of detailed structural data on many enzymes due to difficulties with crystallisation can hinder engineering processes. However the advent of homology modelling,^[5]^ *de novo* structure prediction by AlphaFold,^[6]^ and advancement of machine learning tools in general that only cost a couple of clicks have accelerated enzyme engineering several fold in recent years. However, artificial intelligence based tools still have to prove themselves as an integral part to efficiently complement protein engineering.[^7^, ^8^] Even with sufficient structural data to make informed changes in the protein sequence, positive mutations can come few and far between, and are not always beneficial when added together,^[9]^ whereas practical challenges that are frequently encountered owing to low expression and insufficient protein solubility in vitro are not even considered at this stage. Even though deep learning approaches that assist in identification of mutations can substantially benefit engineering campaigns, the amount of data to be collected to generate a reliable model should not be underestimated and can often impose a bottleneck.^[10]^

In addition, since approximately 99 % of bacteria cannot be cultured in vitro in a laboratory environment due to specific, often unknown, media requirements, temperature, growth factors or a symbiotic host to adhere to,^[11]^ many existing biocatalyst panels are derived only from the remaining 1 %. Nowadays, many companies such as Novozymes or Megazyme provide commercial sources for enzymes and kits to assay biocatalytic reactions, or they produce high quality variants for specific transformations using technology platforms, such as Codexis’ CodeEvolver^®^. Despite thorough analysis of the primary sequence space that can be accessed from current genomics databases and assistance with the search for a better starting enzyme, commercial and open databases (e.g., Uniprot,^[12]^ Brenda,^[13]^ RetroBioCat^[14]^) may still fall short with regard to information on non‐natural enzyme substrates. This shortcoming means it is often necessary to simultaneously screen known biocatalysts along with previously uncharacterised homologues derived from databases, which poses the question “which wild‐type (WT) sequence is the perfect parent for subsequent directed evolution?”

Access to metagenomic libraries, which consist of genetic material recovered from different environments by isolation and sequencing of genomic DNA, frequently reveals significant uncharted sequence space from thousands of previously un‐investigated sources. In addition to this, genome mining grants access to specialised metabolites unseen under generic growth conditions, along with the isolation of desired enzymes from natural product and metabolite pathways, as well as neighbouring enzymes from said pathways that have co‐evolved together.^[15]^ A prerequisite of metagenomics is the availability of standard genomics technologies, including low‐cost sequencing platforms, as well as elaborate bioinformatics tools and software for the processing, filtering, and analysis of large metagenomic datasets.

### Unravelling Sequence Space: Opportunities and Limitations for Biocatalyst Mining

**Figure 2:**
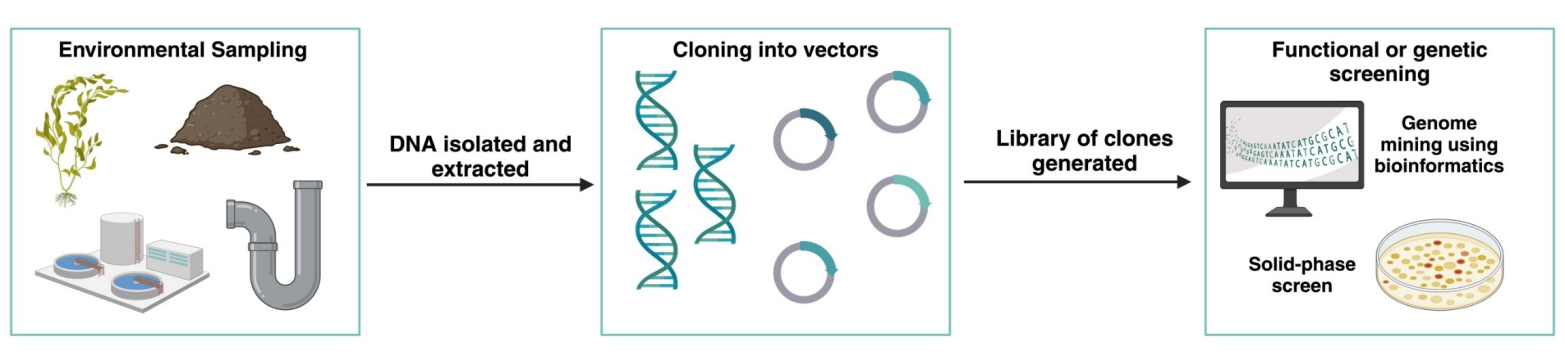
Flowchart representing the general workflow of metagenomics from ‘sample to table’. Samples are collected from different environments and the total DNA is extracted, sequenced, and stored as a library. Bioinformatics tools can then be used to mine novel sequences using known enzymes of interest. Alternatively functional screens such as solid phase assays can be used to identify positive clones, sharing activity in a target transformation.

Formerly, the isolation and sequencing of various environmental samples has been a long and arduous process, as demonstrated with the human genome project. Researchers typically relied on 16S and Sanger sequencing for construction of their metagenomic libraries. Such methods can provide information on the species of the organisms but comes with a caveat of biased sequencing, as genes of interest are amplified by targeted primers, which could subsequently miss the most diverse and novel proteins.[^16^, ^17^] Coupled with the low‐throughput of these methods,^[18]^ large metagenomic libraries will struggle to be thoroughly analysed by 16S and Sanger. The advances made with respect to next‐generation sequencing, especially significant cost reduction, have transformed the field of microbial ecology through metagenomic studies by simplifying the elucidation of an organisms’ genome, making it easier to isolate and characterise genes.^[19]^ The ease with which one can sequence DNA has also paved the way to unprecedented progress in the development of new biocatalytic processes due to a myriad of new enzymes that have become available from hitherto inaccessible sources. Diverse microbiomes that can be unveiled using metagenomics can provide a route to the discovery of novel biocatalysts, expanding sequence space and enabling the identification of enzymes from extreme habitats that can often turn out to be superior for process conditions.^[20]^ However, the exploration and analysis of these data can be somewhat complex and computationally demanding. Moreover, it generally requires previous knowledge of bioinformatics tools and software enabling the processing of large sequence sets. Briefly, a workflow in metagenomics covers the following steps: (i) environmental sampling; (ii) sample processing including DNA extraction,^[21]^ (iii) DNA sequencing, (iv) data processing and statistical analysis including genome mining and using bioinformatics tools to identify sequences of interest (Figure 2).^[22]^

However, sourcing new enzymes comes with restrictions, including the lack of open access and usability of potentially new biocatalysts. The protection of information, such as primary sequence space, can be extremely important in many commercial applications and patents. Therefore, the discovery of novel biocatalysts from private, in‐house metagenomes provides a level of sequence security that may not be gained through conventional genomics. The main reason is that for only a small fraction of all organisms have their genome been determined and deposited in the open database, thus leaving vast areas of sequence space ‘hidden’. Furthermore, sampling parameters such as exact location, seasonal differences, and preparation of genomic material have a severe impact on the composition of the metagenome that consequently results in a ‘uniqueness’ of a metagenomic database. The exploitation of this hidden space can reveal novel biocatalysts, superior to reverse engineering methodologies, such as peptide mass fingerprinting, which struggle to identify exact or even similar matches.

To allow for a fair access to genetic material the Nagoya Protocol on ‘Access to Genetic Resources and the Fair and Equitable Sharing of Benefits Arising from their Utilization (ABS)’ regulates the exploitation of genetic material between different countries.^[23]^ This protocol has a significant impact on the discovery and usage of new enzymes encoded from metagenomes. The protocol was first adopted on 29 October 2010 by the Conference of the Parties to the Convention on Biological Diversity and was fully implemented on 12 October 2014. The Nagoya Protocol on ABS aims to provide fair and justifiable benefits arising from the use of *genetic resources*. However, the complete definition of what constitutes a *genetic resource* is yet to be established, critically missing clarification as to whether this just includes the organism itself, or if it also covers the digital sequence information (DSI) of the organism's genome. The implementation of the protocol prevents access and utilisation of genetic resources without mutually agreed terms being in place that intend to outline the benefit of sharing commitments from both the user and supplier of the resource. There are still grey areas that need to be addressed, such as collaborative working and the publishing of information to online databases, but the inception of such agreements on access and benefit should help combat the issue of biopiracy.

### Harnessing Vast Sequence Space through Screening Methods

**Figure 3:**
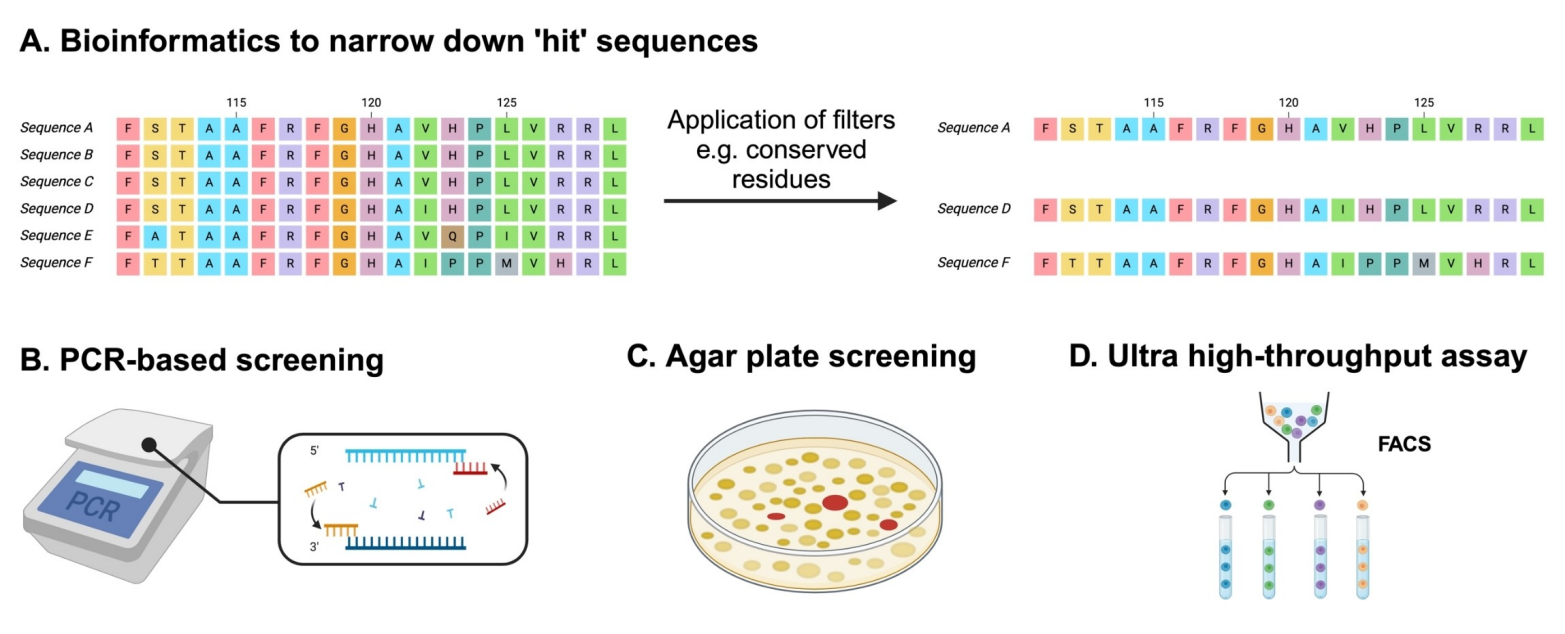
Summary of screening techniques for metagenomic libraries. A: Sequence based bioinformatics tools on online metagenomic databases can help to narrow down hit sequences through the application of filters. B: PCR based screening techniques involve amplification of desired sequences from library by addition of targeted primers. C: Solid phase assay such as agar plate screening provides an efficient way to assay clones. D: Ultra high‐throughput screening such as FACS can sort up to 10^8^ colonies per day.

Metagenomic libraries have traditionally been interrogated through either sequence based or functional screening techniques.^[24]^ Sequence based screening relies on at least some known sequences of the target gene family, which are searched based on homology or motifs, whereas functional based screening does not need such data and usually leads to the discovery of more novel gene sequences in any given sample. Due to the vast majority of microbes being difficult to culture in laboratory conditions as described above, structural knowledge and data on many enzymes is scarce.^[25]^ More importantly, it is estimated that over half of the sequences in open databases have unknown or incorrectly described functions.[^26^, ^27^] These bottlenecks in turn pose a great challenge to acquire efficient sequence based screening methods of huge metagenomic libraries, as usually some knowledge is required as to which type of enzyme is to be searched for. Having access to computational databases provides a shortcut in identifying sequences of interest, allowing sequence‐based screening tools such as bioinformatics to identify hits from conserved regions of target proteins, which narrows down the search for metagenomic enzymes.^[28]^ Activity screening is suitable for smaller libraries as all candidates must be cloned and expressed to be tested against the substrate library of choice. Purified enzyme is always preferred due to removal of side reactions that may occur in the lysate, but this hindrance coupled with expression trials can add time and cost to screening. In addition, poor host expression results in there being very little activity to screen for. Functional screening such as solid‐phase assays of bacterial clones (Figure 3) provides a more rapid assessment of the activity of a library,^[29]^ but ideally being able to filter for redundant sequences before cloning the library would be ideal. By creating digital libraries from the physical DNA samples taken, billions of sequences can be accessed and analysed in any one given query. Enzymes similar to candidates in the open database can be identified by BLAST search using open data sequences of similar function. Hit sequences can then be filtered for the desired similarity and diversity to open data by applying certain criteria such as sequence length, conserved regions or residues, or sequence similarity to other candidates. However, this approach comes with the caveat of potentially missing the discovery of unique enzymes with new chemistries as they may evade sequence homology searching.

In order to assay a large library, (even a reduced library filtered by computational tools can still contain thousands of ‘hit’ sequences), advancements in droplet microfluidics[^30^, ^31^] as well as other advanced screening techniques, for instance, implementation of high‐throughput mass spectrometry can provide rapid in vivo assays of clones.^[32]^

In view of the opportunities for exploiting metagenomic enzymes for sustainable chemical manufacturing, this perspective aims to outline important examples where such enzymes have been utilised in biocatalytic processes. We discuss whether the continuous demand for finding promiscuous biocatalysts for robust processes, as well as the search for better starting points to be applied in engineering campaigns;^[33]^ can be accelerated by metagenome mining.

### Key Metagenomic Enzymes in Biocatalysis

Early examples of the exploitation of metagenomics to access novel sequence space was demonstrated by companies such as Diversa and B.R.A.I.N (now Brain‐Biotech), with both companies pioneering activity screening of metagenomic libraries, since sequencing was expensive and time‐consuming at that time. Initial panels of metagenomic enzymes were generated with nitrilases,^[34]^ carboxylesterases^[35]^ and laccases.,^[36]^ with specific focus on α,β/‐hydrolase enzymes such as lipases, where many reviews have already been published.[^37^, ^38^, ^39^, ^40^, ^41^] A specific advantage of isolating metagenomic enzymes is that they are evolved to the conditions of the habitat from where they are isolated. Such benefits include stability related properties, which can translate directly to biocatalysis applications, as the enzymes are frequently more active under harsh reaction conditions, such as high temperatures and the presence of organic cosolvent, hence making them better suited candidates for chemical processes.

Increased robustness, without the need for protein engineering, is generally favourable for in vitro applications of enzymes where conditions can often lead to rapid enzyme deactivation. In addition to aiding the discovery of novel promiscuous biocatalysts, metagenomics can also serve as a way of gathering genomes of extremophiles from environmental sampling, where the vast majority are intracellular enzymes. For example, cold adapted enzymes have the benefit of lower operating temperatures, reducing environmental pressure and costs to run reactions, while still possessing the activity without the need for cooling.[^42^, ^43^] These enzymes can also be easily inactivated by moderate temperature increases, thus making them ideal for controlling activity in multi‐enzyme reactions, whereas thermostable enzymes can tolerate industrial temperatures without the need for protein engineering. In this next section, we discuss metagenomic enzymes that have been isolated to address the current key reactions in biocatalysis.

### C=X Bond Reduction

Biocatalytic chiral amine synthesis has recently been impacted by metagenomics. Approximately 40 % of pharmaceutical compounds and 20 % of agrochemicals possessing a chiral amine moiety.^[44]^ Enzymes that catalyse the synthesis of these building blocks include (but are not limited to): reductive aminases (RedAms), monoamine oxidases (MAO‐Ns), amine dehydrogenases (AmDHs) imine reductases (IREDs) and transaminases (ATAs).

**Figure 4:**
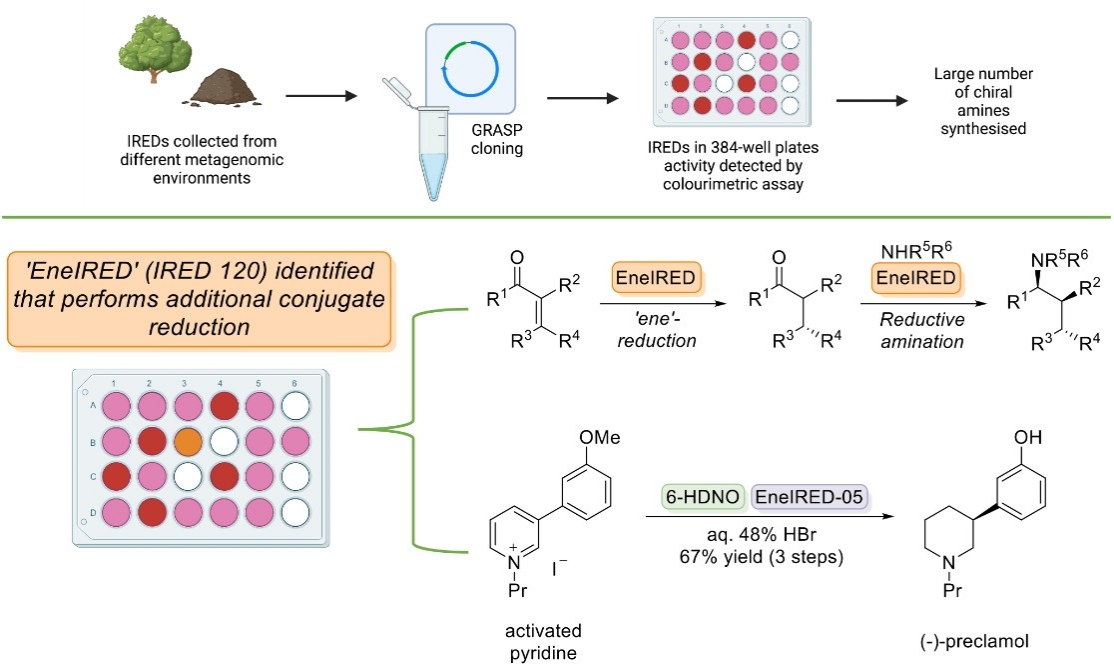
Metagenomic imine reductases were identified from various domestic. U.K. environments in compliance with Nagoya protocol. IREDs could be rapidly screened by fluorescence assay for the transformation of a wide variety of chiral amines.[^50^, ^52^, ^53^] Figure partially created with Biorender.

The application of IREDs for chiral amine synthesis has been aided by the discovery and development of a panel of 384 different IREDs, the vast majority of sequences being derived from UK metagenomic sources. (Figure 4)^[45]^ Marshall et al. also reported an efficient and reliable method for substrate profiling in the oxidative direction by using a colorimetric screen involving NAD(P)H formation to drive reduction of a tetrazolium salt to its corresponding red/pink formazan dye. The application of the metagenomic IREDs was shown to catalyse a variety of aliphatic, aromatic, and heterocyclic ketones with multiple amine donors, as well as β‐keto ester derivatives and several active pharmaceutical ingredients (API) fragments.[^46^, ^47^, ^48^, ^49^] This led to the discovery of an enzyme with the additional capability to catalyse C=C bond reduction as well as reductive amination (EneIRED).^[50]^ It should be noted that previously metagenomic panels of ‘ene’‐reductases have also been discovered and developed (Figure 4).^[51]^

The application of enzymes derived from metagenomes in chemo‐enzymatic as well as fully biocatalytic cascades can lead to an enhanced range of products available for industrial processes. Harwara et al. utilised metagenomic homologues of the newly discovered EneIRED in a chemo‐enzymatic cascade to afford various stereo‐enriched piperidines. (Figure 4)^[52]^ These examples highlight the synthetic value of a metagenomic IRED panel that to date has been successfully exploited for a variety of target reactions. Recently, an *in silico* tool allowing for computational pre‐selection of potential variants based upon a modified IRED library has been implemented.^[54]^ ‘IREDFisher’ utilises homology modelling and molecular docking of target substrates in a web interface, hence ranking IRED homologues based on docking score. Such a combination of metagenomics with bioinformatic modelling offers the potential to further streamline hit identification, with the potential to yield improved success rates to find a suitable enzyme template, e.g., by pre‐screening of large metagenomic datasets.

**Figure 5:**
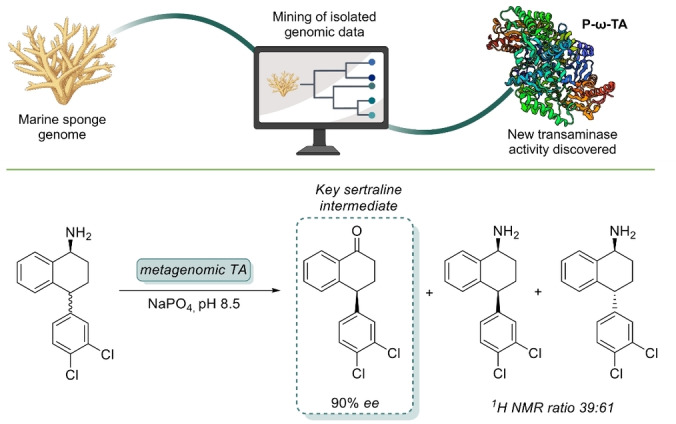
The transaminase P‐ω‐TA was isolated from a marine metagenome and showed increased stability and ability to furnish remote stereoselectivity to form a key intermediate of Sertraline through dynamic kinetic resolution.

Metagenomic transaminases have been found in vastly different environments. Baud et al. discovered a panel of transaminases from an oral microbiome, which displayed good activity towards a variety of different aldehydes and ketones, in particular aromatic substrates.^[55]^ The metagenome of a domestic drainpipe enabled discovery of undescribed, substantially more robust transaminase enzymes.^[56]^ The most promising candidate showed high activity in the presence of 50 equivalents of isopropylamine (IPA), as well as operational stability in 50 % DMSO. Transaminases are usually hampered by their general instability towards higher amine donor concentrations and tolerance to organic solvent, issues that could be overcome in this case by the screening of metagenomic enzymes. O'Gara and co‐workers identified an uncharacterised transaminase from a marine metagenome, which was able to set a second stereogenic centre in the starting material up to 4 bonds away (Figure 5). Most notably one of the substrates is an important precursor to sertraline. Upon examination of the active‐site it was found that the transaminase from *Pseudovibrio* WM33 naturally possessed amino acid substitutions that were introduced into other transaminases to improve reaction rate and melting temperature, showing that metagenomic enzymes can often circumvent tedious engineering efforts.^[57]^

**Figure 6:**
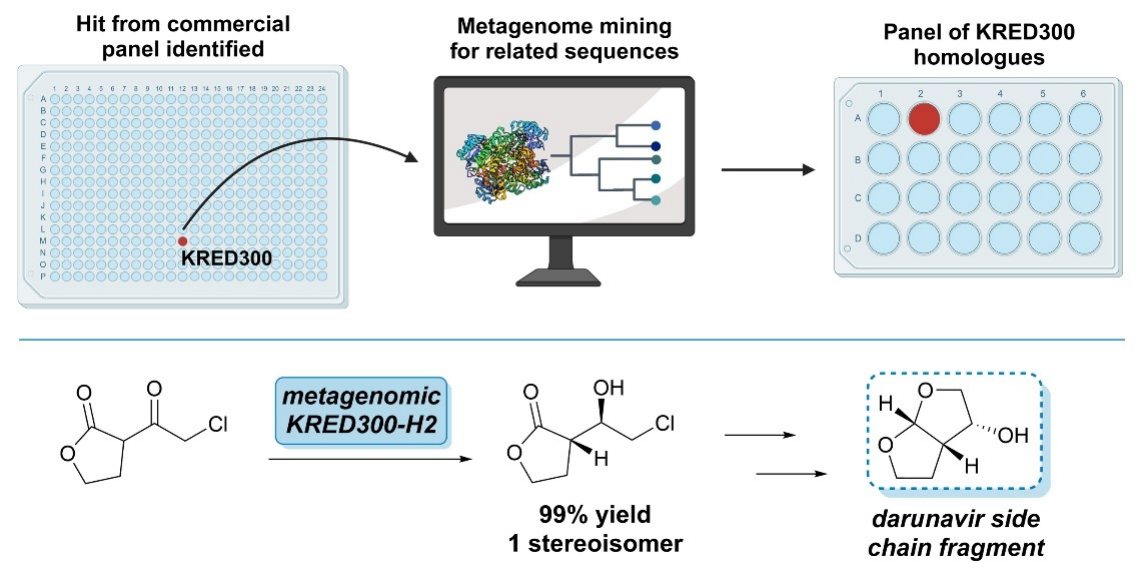
Metagenome mining campaign from a commercial panel of KREDS found novel homologues used to address an early step in the generation of the complex side chain of Darunavir.^[61]^

Asymmetric reduction of carbonyls is an essential transformation in biocatalysis. Ketoreductases (KREDs) or alcohol dehydrogenases (ADHs) are able to the reduction of ketones in a wide range of different molecules.^[58]^ Consequently, many companies have invested in commercial panels of KREDs from known or metagenomic sources along with assays to screen large libraries at once.^[59]^ Newgas et al. isolated a panel of KREDs from an in silico oral microbiome metagenomic library, which demonstrated excellent activity on a number of sterically hindered ketones.^[60]^ It has also been shown that metagenomic KREDs can be used to make synthetically challenging drug fragments. Darunavir is a protease inhibitor drug that treats HIV‐AIDS and possesses a bicyclic acetal side chain with three stereogenic centres. Consequently, the synthesis of the side chain requires harsh conditions and complicated ligands. Riehl et al. used KRED300‐H2 from mining 11 metagenomes using KRED300 as their reference point from the commercial panel of KREDs (Figure 6). After applying a maximum sequence similarity of 95 % to remove the closest homologues, 22 candidates were selected for testing from 21000 genes. KRED300‐H2 was the top performer, allowing generation of the crucial halohydrin intermediate with excellent enantioselectivity and stability in 10 % MeCN, providing a superior route to the bicyclic side chain than standard chemical processes without any protein engineering required.^[61]^

### C−H Activation

**Figure 7:**
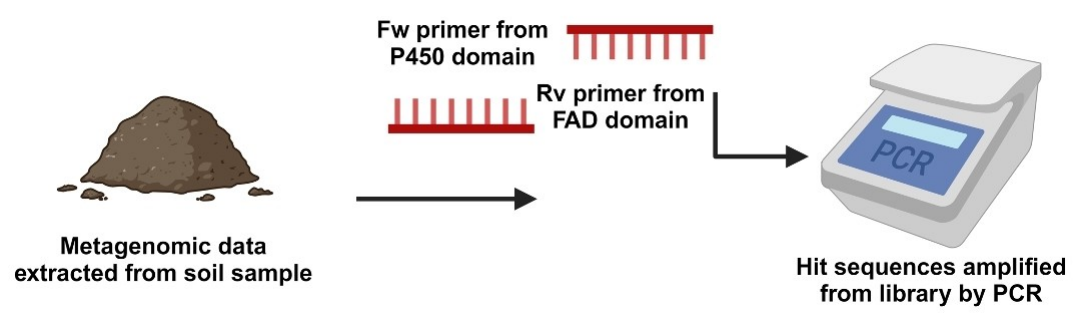
Kim et al. showed that novel metagenomic P450s could be isolated from samples by PCR amplification. P450 SYK181 showed broad substrate scope for hydroxylation including phenanthrene and naphthalene.

Cytochrome P450 monooxygenases are ubiquitous enzymes primarily used for selective oxidation reactions, e.g. hydroxylation^[62]^ and epoxidation^[63]^ including the late‐stage functionalisation of many natural products.[^64^, ^65^] The majority of P450 monooxygenases require a separate ferredoxin reductase for electron shuttling, promoting the search for self‐sufficient P450 enzymes with a tethered FAD/FMN reductase domain. These self‐sufficient enzymes typically have two highly conserved domains (P450 and reductase), and hence the search through metagenomic data can be accelerated via more targeted queries. Kim et al. isolated the DNA from a soil sample and searched for self‐sufficient P450 encoding genes using designed primers based on the known sequence of CYP102 A1.^[66]^ Hit sequences were amplified and SYK181 was successfully purified and showed significant activity towards a number of fatty acids as well as naphthalene and phenanthrene.^[67]^ This example also illustrates the different ways that libraries can be screened if bioinformatics tools are not available. Other examples of the successful isolation of P450 enzymes from metagenomic environments include the discovery of novel thermostable P450s from the Bin Chau hot spring in Vietnam, yielding candidates with a range of high melting temperatures ranging from 50 to >65 °C. (Figure 7)[^68^, ^69^]

**Figure 8:**
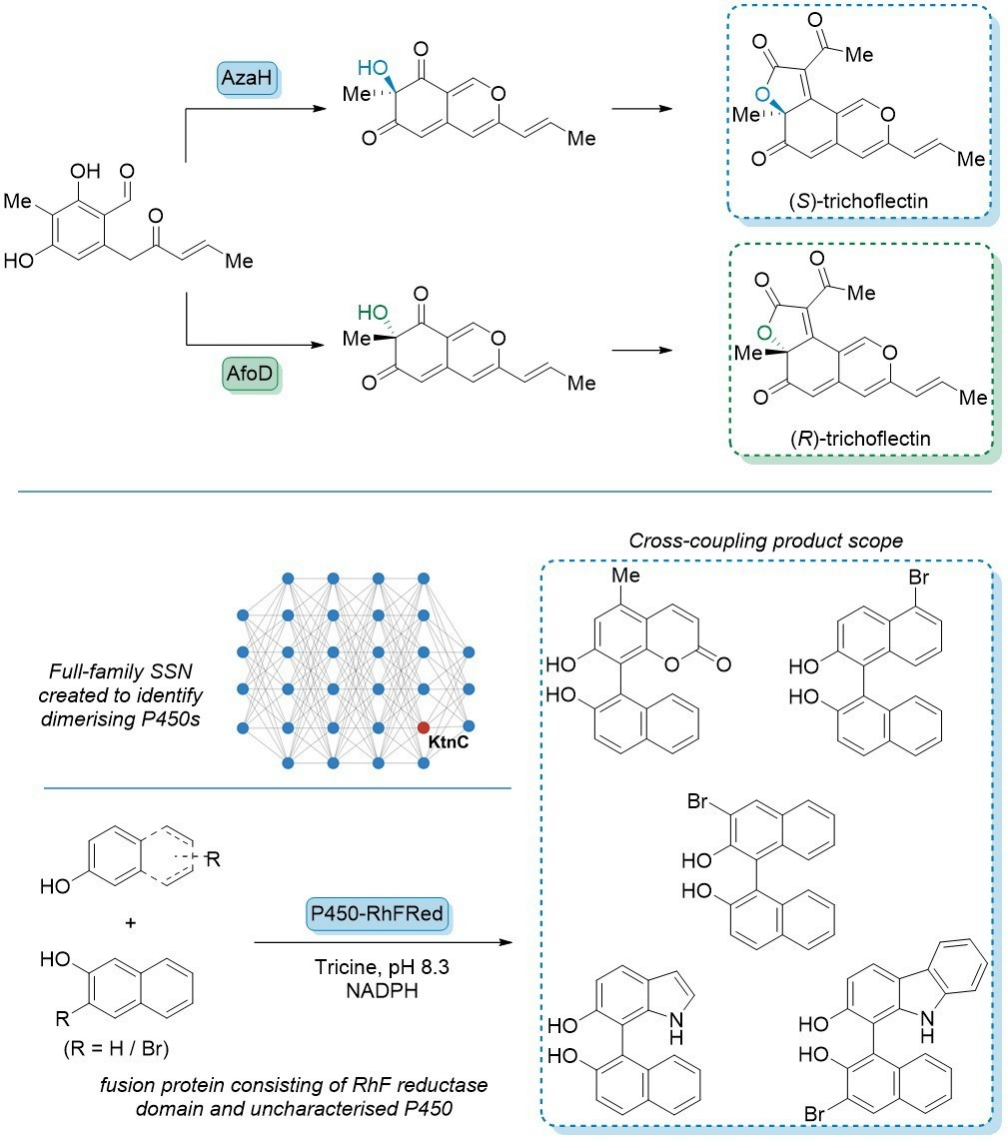
The Narayan group used SSN to discover enantio‐complementary monooxygenases for the synthesis of (*R*)‐ and (*S*)‐ trichoflectin, and for oxidative biaryl cross‐coupling reactions.

More recently a pair of FAD dependant monooxygenases were discovered through sequence similarity network construction, which were capable of oxidative dearomatisation of asperfuranone compounds. AzaH and AfoD discovered by Narayan and co‐workers were isolated by SSN of 45000 oxidative enzymes from the Pfam database. The group demonstrated that using SSNs that combine known and unknown proteins can allow predictions of regio and stereo selectivity from each unique protein cluster verified in these two enantiocomplementary biocatalysts to make (*R*)‐ and (*S*)‐trichoflectin (Figure 8).^[70]^ Bioinformatics tools such as EFI‐EST software can generate a sequence similarity network (SSN) to identify candidates that could carry out oxidative biaryl cross‐coupling reactions. The search revealed 20 uncharacterised proteins, which had activity towards the dimerization of phenolic compounds (Figure 8).[^71^, ^72^]

**Figure 9:**
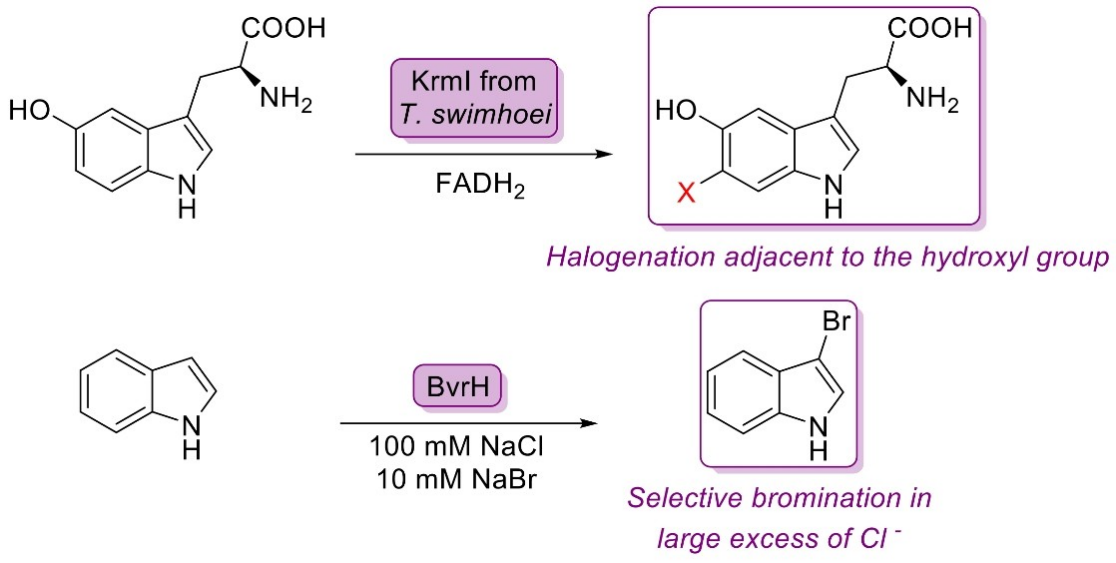
Metagenomic halogenase KrmI allows selective halogenation adjacent to the alcohol group on 5‐HTP. BvrH shows an unusual preference for selective bromination of indole substrates despite large concentrations of chloride.

Halogenase enzymes have emerged as potentially versatile biocatalysts for the late‐stage functionalisation of bioactive molecules,^[73]^ as halogen atoms are crucial for occupying binding sites and improving ligand receptor interactions.^[74]^ A metagenomic flavin‐dependent halogenase KrmI isolated from the metagenome of a marine sponge was discovered to have a preferred substrate of L‐5‐hydroxytryptophan (5HTP) rather than tryptophan.^[75]^ In addition to this, the substrate scope of the halogenase was much broader, with KrmI accepting a range of indole containing substrates as well as serotonin. This promiscuity encouraged further simplified protein engineering efforts to gain access to a wider variety of substrates (Figure 9). Additional studies of marine metagenomes have revealed other interesting candidates such as BrvH, which prefers bromination over chlorination of substituted indoles even in large excesses of chloride (Figure 9).^[76]^

### Enzymatic Hydrolysis

Hydrolase enzymes are ubiquitous in nature and are responsible for the metabolism of large and often xenobiotic molecules.^[77]^ Consequently, their uses in industry and specifically waste management are advantageous. Industrial conditions usually require robust enzymes that are able to tolerate harsher conditions than in a research lab, to aid solubility of compounds and speed up reaction rates. Therefore, many hydrolases that are discovered (mostly esterases) come from more extreme environments.[^78^, ^79^, ^80^]

The Ward group discovered a new set of epoxide hydrolase (EH) enzymes from a soil metagenome, which was chosen due to previously isolated EHs displaying activity on soil contaminants.^[81]^ Mining their in‐house database of over 42,000 metagenomic sequences led to new 3 limonene epoxide hydrolases (LEHs) and 30 α/β‐EH sequences being identified and characterised that accepted a wide variety of aromatic, aliphatic and *meso*‐epoxides. Interestingly, one candidate enzyme had comparable activity in the presence of 5–30 % MeOH still forming the diol rather than the methoxy ether, ideal for industrial applications and overcoming issues such as substrate solubility at high concentrations. α/β‐EHs have also been identified from mangrove soil metagenomes, using conserved residues across the enzymes family as search criteria applying Hidden Markov models.^[82]^ LEHs have also been discovered from a hot spring metagenome, and have thus demonstrated remarkable thermostability with operational T_m_’s of up to 74 °C.[^39^, ^40^, ^41^, ^83^]

With polyethylene terephthalate (PET) being the most common plastic, being produced on a hundred million‐tonne scale, the demand for novel plastic degrading enzymes that can break down PET has never been greater.^[84]^ Metagenomics could be key in solving the problem of plastic accumulation that society is currently facing, as the most thermostable wt PETase discovered is from a compost metagenome^[85]^ and has been successfully engineered in a number of campaigns to elevate its stability and activity.[^86^, ^87^, ^88^] Other PETases have since been discovered in a human saliva metagenome and successfully engineered via genetic code expansion to tether protein cargos to the plastic surface for wearable disease biomarker detection.^[89]^ There is now a focus on the degradation of polyurethanes and polyamides in the environment with the first of these enzymes being discovered from soils exposed to these chemicals.^[90]^ As the planet's environment changes, so will the nature of the microorganisms in them. There is now evidence that plastics and other toxic compounds have leached into the environment and are causing selection pressures, with bacteria evolving to perform better with these new carbon sources. One way this can occur is through alterations in the enzymes (gene/protein sequence) that degrade these novel polymers.^[91]^ This has already lead to the discovery of more PETases as well as other plastic degrading enzymes being present in the composition of different environments and these enzymes can be harnessed to accelerate plastic recycling and solve timely challenges that the world is facing.^[92]^

## Future Perspective

One remaining question is ‘how much sequence space is available?’ Kyrpides and co‐workers recently demonstrated the power of using computational approaches to find proteins in sequence space that has never been explored, isolating and sorting through more than 27000 metagenomic datasets to generate the novel metagenomic protein family (NMPF) database, comprised of over 106,000 protein families with no sequence similarity to proteins in the current PFam database. The total number of sequences longer than 35 amino acids estimated to be over 1 billion.^[93]^ This establishes the precedence for novel databases to be created such as FESnov^[94]^ which is comprised of over 400000 gene families from uncultivated prokaryotes. With all of this microbial ‘dark matter’ yet to be uncovered,^[93]^ and further advancement in collecting and sequencing samples, new proteins will be found faster than ever that could advance a plethora of different industries. As more sequences are discovered, the challenges aring from data processing, sorting and annotating will increase. However, advancements in technology and data storage, as well as knowledge around protein sequences, should hopefully keep pace with the rate of biocatalyst discovery.

## Conclusion

Metagenome mining allows the general and targeted roaming of sequence space for a particular target catalyst in previously unexplored and unique sequence space, without applying several rounds of evolution. This approach offers a complementary solution to finding novel biocatalysts for sustainable chemistries. Metagenomics can potentially provide researchers with a much better and more optimised starting point for evolution towards a promiscuous biocatalyst. Furthermore, the increase in sequence knowledge of an enzyme class, e.g., conserved residues can facilitate easier and faster evolution to afford more specialised enzymes. Overall, we envision that metagenomics at its core will continue to remain a powerful tool for biocatalysis in the years to come.

## Conflict of interests

The authors declare no conflict of interest.

1

## Biographical Information

*Bethany N. Hogg received her MChem in Chemistry with Medicinal Chemistry in 2019 from the University of Manchester. She pursued her Ph.D. studies under the supervision of Prof. Nicholas Turner where she is now a postdoctoral research associate, studying metagenomic enzymes for the synthesis of 1,2‐bifunctional intermediates. Her research interests include biocatalysis, specifically metagenomics and protein engineering*.

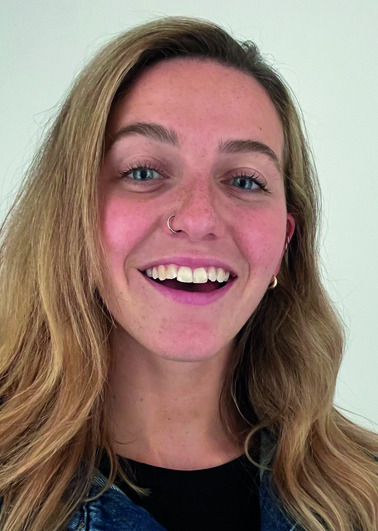

## Biographical Information

*Christian Schnepel is Assistant Professor of Biocatalysis at KTH Royal Institute of Technology (Sweden). He studied biochemistry focusing on Chemical Biology, receiving his M. Sc. degree in Biochemistry from Bielefeld University. In 2019, he completed his PhD studies on enzymatic halogenation under the supervision of Prof. Norbert Sewald and obtained the Max‐Bergmann Young Investigator Award. After his postdoctoral stay with Prof. Nicholas J. Turner at Manchester Institute of Biotechnology (UK), he moved to Sweden to start his independent career. His research interests include biocatalysis, enzyme engineering, and cascades for fine chemical synthesis and pharmaceutical chemistry*.

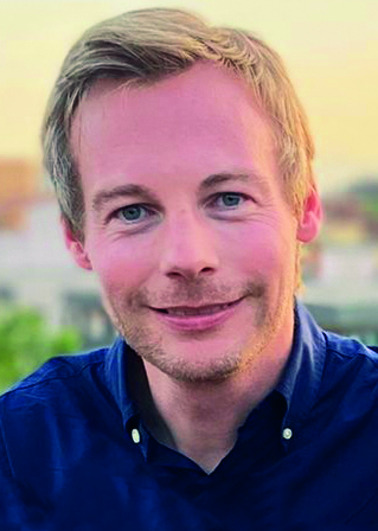

## Biographical Information

*James D. Finnigan is currently Technical Director of Prozomix Limited. He joined Prozomix during his Ph.D. studies in 2014 under the supervision of Prof. Gary Black at the University of Northumbria, working on the discovery of novel metagenomic P450s. Following his studies, he continued his work in enzyme discovery from in‐house metagenomics libraries, from a variety of different enzyme families, across a number of UK and EU funded projects*.

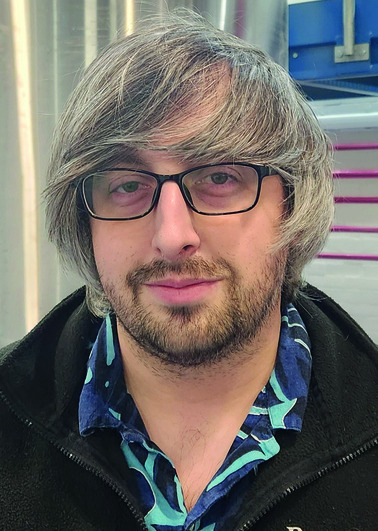

## Biographical Information

*Simon J. Charnock is currently CEO of enzyme discovery and production company Prozomix Limited, that he co‐founded in 2008. He completed his Ph.D. in 1998 under the supervision of Prof. H. J. Gilbert FRS at the University of Newcastle. After postdoctoral studies at the University of York with Prof. Gideon Davies FRS, he briefly lectured before co‐founding and heading‐up the Molecular Biology Division at Megazyme International, before co‐founding Prozomix. Since 2008, he has guided the company to become a leading provider of novel biocatalysts focused on pharma applications*.

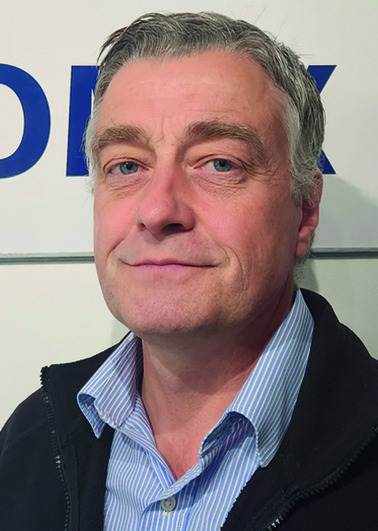

## Biographical Information

*Martin A. Hayes is currently Biocatalysis Leader at the iLAB, Discovery sciences at AstraZeneca, Gothenburg. He completed his PhD in 1991 with Prof. T. J. Simpson FRS at the university of Bristol. After postdoctoral studies at the University of Toronto with J. Bryan Jones, he started his industrial career. He has contributed to the discovery of many small molecule therapeutics including Brilinta and the FLAP inhibitor AZD5712. His research interests include biocatalysis, drug design and high‐throughput experimentation*.

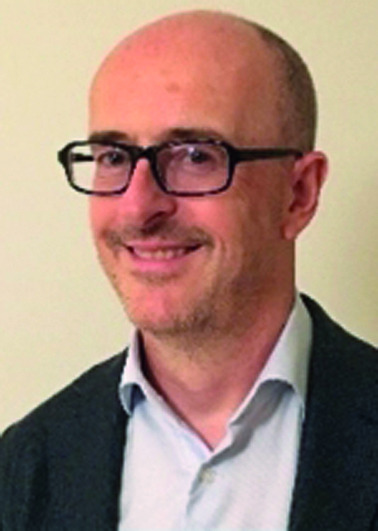

## Biographical Information

*Nicholas J. Turner FRS is Professor of Chemical Biology at the University of Manchester in the Manchester Institute of Biotechnology. His research interests are in the area of biocatalysis with particular emphasis on the discovery and development of novel enzyme‐catalysed reactions for applications in organic synthesis. His group have published >400 papers and developed engineered biocatalysts which have found wide application in synthesis*.

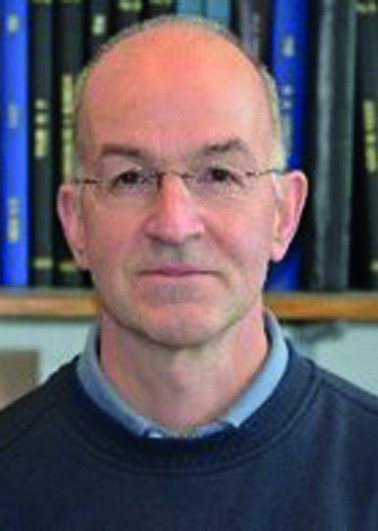

## Data Availability

Data sharing is not applicable to this article as no new data were created or analyzed in this study.
